# The frequency of *NUDT15* rs116855232 and its impact on mercaptopurine-induced toxicity in Syrian children with acute lymphoblastic leukemia

**DOI:** 10.3389/fonc.2024.1334846

**Published:** 2024-03-18

**Authors:** Muhammad Muhammad, Maher Saifo, Majd Aljamali, Mousa Alali, Khaled M. Ghanem

**Affiliations:** ^1^ BASMA Pediatric Oncology Unit, Damascus, Syria; ^2^ Department of Oncology, Albairouni University Hospital, Faculty of Medicine, Damascus University, Damascus, Syria; ^3^ Faculty of Medicine, Damascus University, Damascus, Syria; ^4^ Department of Biochemistry and Microbiology, Faculty of Pharmacy, Damascus University, Damascus, Syria; ^5^ National Commission for Biotechnology (NCBT), Damascus, Syria

**Keywords:** *NUDT15*, genotype, acute lymphoblastic leukemia, mercaptopurine, toxicity, Syria, pediatric, pharmacogenetics

## Abstract

**Introduction:**

Polymorphisms in *NUDT15* may result in differences in mercaptopurine-induced toxicity. This study aimed to identify the frequency of the *NUDT15* (c.415C>T; rs116855232) polymorphism and investigate the effect of this polymorphism on mercaptopurine-induced toxicity in a population of Syrian patients with childhood acute lymphoblastic leukemia (ALL).

**Methods:**

This is a retrospective study that included children with ALL reaching at least 6 months of maintenance therapy. The *NUDT15* genotyping was determined using standard targeted sequencing of polymerase chain reaction products. The odds ratio (OR) for the association between toxicity and genotype was evaluated.

**Results:**

A total of 92 patients were enrolled. The majority of the patients in the study population were low-risk (63.04%), followed by intermediate-risk (25%), and high-risk (11.96%). There were 5 patients (5.4%) with *NUDT15* (c.415C>T; rs116855232) CT genotype, and 1 patient (1.08%) with *NUDT15* TT genotype, with allele frequencies of C=0.962 and T=0.038. The mercaptopurine median dose intensity was 100%, 54.69%, and 5% for the genotypes CC, CT, and TT, respectively (*P*=0.009). Early onset leukopenia was significantly associated with the *NUDT15* polymorphism (OR: 6.16, 95% CI: 1.11-34.18, *P*=0.037). There was no association between the *NUDT15* genotype and hepatotoxicity.

**Conclusion:**

Approximately 6.5% of the study population exhibited reduced NUDT15 activity. The mercaptopurine dose intensity was considerably low in *NUDT15* rs116855232 TT genotype compared with CT and CC. The dosage of mercaptopurine should be adjusted according to the *NUDT15* genotype in pediatric patients with ALL.

## 1 Introduction

Approximately one third of all cancers in children are acute lymphoblastic leukemia (ALL) (1–3), which can be cured by chemotherapy alone (4).

In pediatric patients with ALL, the antimetabolite prodrug 6-mercaptopurine (6MP) is a key chemotherapeutic agent for maintenance therapy. In addition, it is widely used in various phases of ALL treatment (5). 6MP needs to be metabolized to thioguanosine triphosphate (TGTP) which is incorporated into DNA to form a DNA-TG compound, causing futile DNA damage that leads to cell apoptosis (6, 7).

Specific polymorphisms in genes involved in the metabolism of thiopurine can directly affect toxicity and effectiveness. There is evidence that several single nucleotide polymorphisms (SNPs) can inhibit thiopurine metabolism enzyme activity, causing thioguanine nucleotide accumulation and hematologic toxicity. Nucleoside diphosphate-linked moiety X-type motif 15 (NUDT15) is a negative regulator nucleotide di-phosphatase that converts TGTP to thioguanosine monophosphate (TGMP), thereby inactivating thiopurine metabolites (8–11).

NUDT15 enzyme is encoded by the *NUDT15* gene, which is located on chromosome 13q14.2 and contains three exons. NUDT15 is a 164 amino acid protein, a member of the Nudix hydrolase superfamily. The *NUDT15* gene was previously sequenced in 6MP-treated patients to identify the reason for significant variability in tolerated dose in patients with similar clinical characteristics treated with the same treatment plan. More than 20 variants of the *NUDT15* gene have been reported to date (12). Polymorphism in *NUDT15* rs116855232 (c.415C>T), which is the core allele for NUDT15*3 leads to changing arginine 139 to cysteine (R139C). This change causes protein instability and decreases NUDT15 activity, leading to accumulation of thioguanine and increased 6MP-related toxicity (9, 13).

Previous studies concluded that the presence of *NUDT15* rs116855232 polymorphism increased the chance of severe leukopenia related to 6MP treatment (14, 15). Therefore, the *NUDT15* genotype can be used to guide the 6MP dose for ALL therapy. This illustrates how pharmacogenetics is driving precision medicine in cancer.

Syria is located on the eastern shore of the Mediterranean Sea in the Middle East. Syria’s location has led to the extensive admixture of its inhabitants with other populations, this has resulted in a complex ethnic, and genetic diversity. The allele frequency of *NUDT15* variants differs between populations. It is 10% in East Asians, 7% in South Asians and Latinos and less than 1% in non-Finnish Europeans and Africans (16, 17). Many studies have examined the impact of *NUDT15* allelic variants in East Asians and other ethnic groups, but they have been scarce in Arabs and the Middle East (18). This study aimed to investigate the frequency of the *NUDT15* (c.415C>T; rs116855232) low-function variant in a population of Syrian children with ALL and to assess the association of this variant with 6MP-induced toxicity and tolerated dose.

## 2 Materials and methods

### 2.1 Ethics statement

The study protocol was approved by the Institutional Review Board of Damascus University, and the committee’s reference number was 514 on December 1, 2019. As per the ages and conceptual abilities of the patients, informed consent was obtained either from the patient’s parents or guardians or from the patients themselves.

### 2.2 Study design and patient eligibility

This is a retrospective cohort study performed at two recruitment sites; BASMA Pediatric Oncology Unit and Pediatric Department at Albairouni University Hospital. Both centers are located in Damascus, Syria. These two centers receive more than 50% of pediatric patients with ALL in Syria. The inclusion criteria were as follows: age less than 19 years, all pediatric patients with ALL who reached at least 6 months of maintenance therapy between January 2018 and May 2020. Patients with hepatic or renal failure, life-threatening diseases, or cancers other than ALL were excluded. A total of 92 patients met the inclusion criteria. Most patients were still receiving maintenance therapy when blood samples were collected, while the rest had finished treatment and were being monitored.

### 2.3 Treatment protocol and measurement of clinical factors

Patients with ALL are classified into three risk categories: Low-risk patients include patients aged from 1 to 9.9 years with B-cell precursor and white blood cell count less than 50 × 10^9^/L and more than or equal to 1.16 DNA index, or *TEL-AML1* fusion, and patients with bone marrow blasts less than 5% at days 19 or 26 or minimal residual disease (MRD) less than 0.01% at the end of induction. The intermediate-risk patients were aged more than or equal to 10 years with T cell or more than 50 × 10^9^/L or B cell with CNS or testicular leukemia, less than 45 chromosomes (hypodiploid), *MLL* rearrangement, or *E2A-PBX1* fusion or patient with bone marrow blasts ≥ 5 at days 19 or 26 or MRD < 1 ≥ 0.01% at the end of induction and patients that did not meet low- or high-risk criteria. The high-risk group includes those with t(9;22) *BCR-ABL* fusion or patients with induction failure (bone marrow blasts more or equal 5%) or MRD ≥1% at the end of induction or MRD ≥ 0.1% at week 7 (19).

Children with low and intermediate risk disease were treated using the European Organization for Research and Treatment of Cancer (EORTC) 58951, average risk-1 (AR1) and AR2 protocols, respectively (20). Children with high-risk disease were treated using the St Jude total XV protocol (21).

The standard 6MP oral dosage during the maintenance phase was 50 mg per m^2^ per day in the EORTC 58951 protocols. In the St Jude Total XV protocol, the standard 6MP was 50 mg per m^2^ per day during the first 20 weeks of maintenance, and 75 mg per m^2^ per day as of week 21 of maintenance.

Clinical history and examination, AEs according to the common terminology criteria for adverse events (CTCAEs) v5.0 (22), and blood tests for blood cell count (CBC), alanine aminotransferase (ALT), and aspartate aminotransferase (AST) were recorded at each visit. 6MP dose intensity, early-onset leukopenia, and treatment interruption due to chemotherapy toxicity were also collected.

As per the protocol, the 6MP dose intensity was determined by dividing the final tolerated 6MP dose by the prescribed 6MP maintenance dose. The 6MP dose was adjusted to maintain the white blood cell (WBC) above 2000 per μL (less than CTCAE G3) and the platelet count above 50.000 per μL (less than CTCAE G3). 6MP treatment interruption was defined as the cessation of chemotherapy administration resulting from infections with or without neutropenia. CTCAEs G3/4 were defined as severe toxicity at any time during maintenance treatment. When leukopenia occurs within 60 days of a maintenance therapy regimen, it is classified as early-onset leukopenia.

### 2.4 Genetic analyses

The total genomic DNA from peripheral blood was extracted using a blood DNA preparation kit (Jena Bioscience, Germany) as instructed by the manufacturer. DNA was stored at -20°C until sequencing. The total genomic DNA concentration was determined using a spectrophotometer (MAESTROGEN, MaestroNano, ProMN-913A, Taiwan). PCR and Sanger sequencing were used to determine the genotype of rs116855232. The sequences of the forward and reverse primers reported in previous publications were 5’-AAGCAAATGCAAAGCATCAC-3’ and 5’-GGCTGAAAGAGTGGGGGATA-3’, respectively (9). Each PCR amplification reaction contained approximately 20 ng DNA, 0.5 µM final concentration of each primer, 10 µl of direct PCR 2 X Master Mix (KAPABIOSYSTEMS, KAPA2G, KR0374-v11.23) and nuclease-free water to a final volume of 20 µl. The reactions were amplified using a DNA thermocycler (Labcycler SensoQuest, Germany). The PCR was performed under the following conditions: initial denaturation at 95°C for 5 minutes, 35 cycles of denaturation at 95°C for 30 seconds, annealing at 52°C for 30 seconds and extension at 72°C for 1 minute, followed by a final extension at 72°C for 10 minutes. An amplicon of 450 bp that contained the rs116855232 site was amplified. DNA extraction and PCR were performed at the Department of Pharmaceutical Biotechnology of the National Commission for Biotechnology, Damascus, Syria. The specimens were stored at -20°C until sequencing at Macrogen Inc. (Seoul, Republic of Korea). Sequencing of the PCR product allowed for the investigation of 4 adjacent polymorphisms to the target SNP (**Table 1**). The results were analyzed using Chromas 2.6 software (Technelysium, Australia) and verified by manual inspection of at least two individuals to ensure accuracy.

**Table 1:** *NUDT15* variants investigated in 450 bp-amplicon PCR product throughout sequencing in 92 pediatric acute lymphoblastic leukemia cases.

Variant	Star alleles	GRCh38 (NC_000022.11)	dbSNP	Variant Impact
**c.386C>G**	*20	g.48045690C>G	rs768324690	P129R
**c.415C>T**	*3	g.48045719C>T	rs116855232	R139C
**c.416G>A**	*4	g.48045720G>A	rs147390019	R139H
**c.467T>A**	*15	g.48045771T>A	rs139551410	L156Q
c.*7G>A	*1.005	g.48045806G>A	rs61973267	No Change

PCR, polymerase chain reaction; SNP, single nucleotide polymorphism; rs, reference SNP.

### 2.5 Statistical analysis

For numeric variables, data were collected and summarized by descriptive statistics and provided as the mean and standard deviation (SD) for normally distributed data variables or as median and range for nonnormally distributed data variables. For nominal variables, data were summarized using descriptive statistics and presented as percentages and frequencies. The frequency of each genotype was calculated using the Hardy-Weinberg equation (HWE) and was determined using the chi-square test. The chi-squared or Fisher’s exact test was used to determine whether there is a statistical association between categorical variables, such as the frequency of dose reductions or interruptions, and SNPs. Genotypes of *NUDT15* were used to categorize patients. For comparing 6MP dose intensity within *NUDT15* genotype categories (independent samples), Kruskal–Wallis nonparametric tests were performed. The effect of rs116855232 on 6MP-induced toxicity was investigated by calculating the odds ratio (OR) and confidence interval (95% CI) by performing logistic regression analyses. SPSS (version 24) was used to perform all statistical analyses. Differences were considered statistically significant if the two-sided *P*<0.05.

## 3 Results

### 3.1 Patient characteristics and genetic polymorphisms

The inclusion criteria were met by 92 pediatric patients with ALL. The patients’ ages at diagnosis ranged from 0.9 to 16 years, and the median age was 5.3 years. Most patients were male (53 patients, 57.61%). Precursor B was the most common immunologic subtype (69 patients, 75%). Low risk was the most common risk group (58 patients, 63.04%). There are statistically significant differences between the *NUDT15* genotypes with regards to leukopenia G3/4 [(37 patients (43%) for CC vs 5 patients (100%) for CT vs 1 patient (100%) for TT; *P*=0.007] and the dose intensity<60% [(20 patients (23.3%) for CC vs 4 patients (80%) for CT vs 1 patient (100%) for TT; *P*=0.005], but there are no differences between them in the other characters. The BASMA Pediatric Oncology Unit and Pediatric Department at Albairouni University Hospital receive more than 50% of children with cancer in Syria. Therefore, the patients who participated in this study came from all provinces around Syria. The demographics and clinical characteristics of the patients are summarized in **Table 2**. The patients in the study population were distributed according to the *NUDT15* c.415C>T (rs116855232) among the majority of wild-type (CC) (86 patients, 93.4%), followed by heterozygous (CT) (5 patients, 5.4%), and a minority (1 patient, 1.08%) of homozygous for the variant (TT). The minor allele frequency (MAF) of *NUDT15* c.415C>T (rs116855232) was 3.8% (**Table 3**). The frequency of the other investigated SNPs is zero.

**Table 2:** Characteristics of patients with ALL according to *NUDT15* genotype (92 patients).

			*NUDT15* (rs116855232, c.415C>T)			
		Total patients N (%)	CC	CT	TT	*P* value*
**Total patients N (%)**			86 (93.4)	5 (5.4)	1 (1.08)	
**Sex**	Female	39 (42.39)	35 (40.7)	3 (60)	1 (100)	0.366
	Male	53 (57.61)	51 (59.3)	2 (40)	0 (0)	
**Age (Year)**	Median (range)	5.3 (0.9-16)	5.4 (0.9-16)	4.01 (3.1-5.6)	5 (NA)	0.342
**BSA (m^2^)**	Median (range)	0.77 (0.52-1.73)	0.79 (0.52-1.73)	0.77 (0.6-0.83)	0.71 (NA)	0.612
**Immunologic subtype**	B-lymphoblastic lymphoma	7 (7.61)	7 (8.1)	0 (0)	0 (0)	>0.999
	T-ALL	10 (10.87)	10 (11.6)	0 (0)	0 (0)	
	T-lymphoblastic lymphoma	6 (6.52)	6 (7)	0 (0)	0 (0)	
	Precursor B	69 (75)	63 (73.3)	5 (100)	1 (100)	
**Risk group (protocol)**	Low risk (EORTC AR1)	58 (63.04)	52 (60.5)	5 (100)	1 (100)	0.557
	Intermediate risk (EORTC AR2)	23 (25)	23 (26.7)	0 (0)	0 (0)	
	High risk (St Jude high-risk)	11 (11.96)	11 (12.8)	0 (0)	0 (0)	
**Leukopenia G3/4**	No	49 (53.26)	49 (57)	0 (0)	0 (0)	**0.007**
	Yes	43 (46.74)	37 (43)	5 (100)	1 (100)	
**Days to first leukopenia G3/4 toxicity**	Median (range)	84 (20-457)	101 (20-457)	63 (42-69)	42 (NA)	0.194
**Thrombocytopenia G3/4**	No	86 (93.5)	80 (93)	5 (100)	1 (100)	>0.999
	Yes	6 (6.5)	6 (7)	0 (0)	0 (0)	
**Days to first thrombocytopenia G3/4 toxicity**	Mean ( ± SD)	168.2 ± (145.18)	168.2 ± (145.18)	NA	NA	NA
**AST/ALTG3/4**	No	80 (87)	75 (87.2)	5 (100)	0 (0)	0.144
	Yes	12 (13)	11 (12.8)	0 (0)	1 (100)	
**Therapy interruption**	No	65 (70.65)	61 (70.9)	3 (60)	1 (100)	0.739
	Yes	27 (29.35)	25 (29.1)	2 (40)	0 (0)	
**6MP dose intensity<60%**	No	67 (72.83)	66 (76.7)	1 (20)	0 (0)	**0.005**
	Yes	25 (27.17)	20 (23.3)	4 (80)	1 (100)	

*P-values were generated by Fisher exact test, chi-squared, and Kruskal–Wallis nonparametric tests as applicable.

ALT, alanine aminotransferase; AST, aspartate aminotransferase; EORTC, European Organisation for Research and Treatment of Cancer; MP, mercaptopurine; NUDT15, nucleoside diphosphate-linked moiety X-type motif 15; SD, standard deviation; ALL, acute lymphoblastic leukemia; BSA, body surface area; AR, average risk; NA, not applicable.The bold values indicate statistical significance (*P*<0.05).

**Table 3:** Frequencies of the identified SNP, genotype data and results of Hardy-Weinberg Equilibrium (HWE) in 92 pediatric acute lymphoblastic leukemia cases.

SNP	%	Genotype	Overall patients (n=92)		
			Observed N (%)	Expected %	HWE *P*-value
**rs116855232,** **(c.415C>T)**	3.8	CC	86 (93.4)	92.5	**0.010**
		CT	5 (5.4)	7.3	
		TT	1 (1.08)	0.1	

HWE, Hardy-Weinberg equation, SNP, single nucleotide polymorphism.The bold values indicate statistical significance (*P*<0.05).

A chi-square test of HWE was performed to compare the observed and predicted frequencies of the genotypes based on the investigated allelic frequency. The balance showed a deviation in the observed frequency of the genotypes of the *NUDT15* c.415C>T (rs116855232) polymorphism from the expected frequency, as there was an increase in the frequency of the heterozygous genotype against a decrease in the frequency of the WT and homozygous genotypes (*P*=0.010) (**Table 3**).

### 3.2 Adverse events during maintenance therapy

At least one AE occurred in most patients (84 patients, 91.3%). Severe AE (G3/4) occurred in half of the patients, most of which were leukopenia (43 patients, 46.7%) and elevated liver enzymes (12 patients, 13%). Treatment was discontinued until toxicity improved in more than a quarter of patients (27 patients, 29.3%) (**Table 4**).

**Table 4:** 6MP-induced toxicity during childhood ALL maintenance therapy (92 patients) N (%).

AE	All Grades	Grade 1	Grade 2	Grade 3	Grade 4
Leukopenia	74 (80.4)	10 (10.9)	21 (22.8)	36 (39.1)	7 (7.6)
Anemia	45 (48.9)	30 (32)	10 (10.9)	5 (5.4)	0 (0)
Thrombocytopenia	25 (27.1)	11 (12)	8 (8.7)	5 (5.4)	1 (1.1)
Elevated AST/ALT	42 (45.6)	15 (16.3)	15 (16.3)	11 (11.9)	1 (1.1)
Vomiting/nausea	4 (4.3)	1 (1.1)	3 (3.3)	0 (0)	0 (0)
Diarrhea	2 (2.2)	0 (0)	2 (2.2)	0 (0)	0 (0)
Alopecia	6 (6.6)	3 (3.3)	3 (3.3)	0 (0)	0 (0)
Pneumonia	1 (1.1)	0 (0)	1 (1.1)	0 (0)	0 (0)
Rash	2 (2.2)	1 (1.1)	1 (1.1)	0 (0)	0 (0)
AEs any grade	84 (91.3)	NA	NA	NA	NA
AEs G3/4	46 (50)	NA	NA	NA	NA
Therapy interruption	27 (29.3)	NA	NA	NA	NA

AEs, Adverse events; ALL, acute lymphoblastic leukemia; ALT, alanine aminotransferase; AST, aspartate aminotransferase; MP, mercaptopurine; NA, not applicable.

### 3.3 The relationship between *NUDT15* genetic variants and 6MP dose intensity

**Figure 1:**
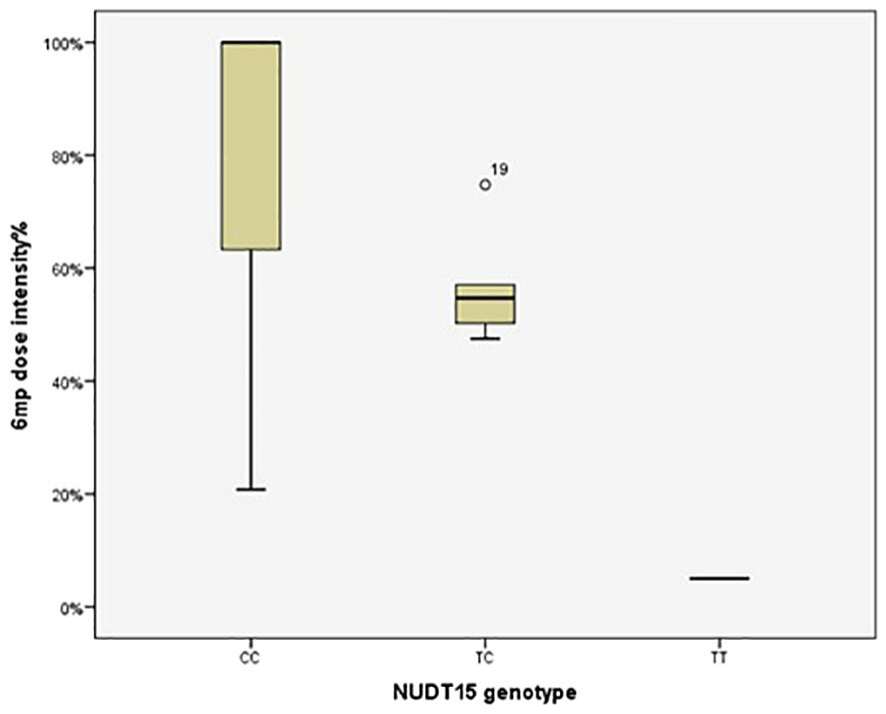
Therapeutic dose of 6-mercaptopurine during maintenance therapy according to *NUDT15* rs116855232 genotype in 92 pediatric acute lymphoblastic leukemia cases. The median 6MP dose intensity was 5%, 54.69% and 100% for the genotypes TT (n=1), CT (n=5) and CC (n=86), respectively (*P*=0.009). *P* value was estimated by the Kruskal–Wallis test.

The 6MP dose intensity was considerably lower in the TT genotype than in the CT and CC genotypes (*P*=0.009) (**Table 5**, **Figure 1**).

**Table 5:** Comparison of 6MP dose intensity % by *NUDT15* genotype in 92 pediatric acute lymphoblastic leukemia cases.

		6MP dose intensity%		
		Median	Minimum-maximum	*P*-value*
*NUDT15*	CC (n=86)	100	21-100	**0.009**
	CT (n=5)	54.69	47-75	
	TT (n=1)	5	NA	

*Kruskall Wallis test.

MP, mercaptopurine; NA, not applicable; NUDT15, nucleoside diphosphate-linked moiety X-type motif 15.The bold values indicate statistical significance (*P*<0.05).

### 3.4 Association between *NUDT15* variants and 6MP toxicity

Early onset leukopenia was observed in 15 patients (16.3%). Leukopenia G3/4 (<2000/mm^3^) at any stage of maintenance therapy was noticed in 43 patients (46.7%). *NUDT15* T allele carriers (CT and TT combined) had a 6.16-fold increased risk of early-onset leukopenia G3/4 compared to patients with WT (CC) (*P*=0.037) (**Table 6**).

**Table 6:** Associations between *NUDT15* genotype and risk of severe adverse events and therapy interruption in 92 pediatric acute lymphoblastic leukemia cases.

		*NUDT15* genotype				
		CC (n=86) N (%)	CT & TT (n=6) N (%)	*P*-value*	OR*	95%CI
**Leukopenia G3/4**	No	49 (57)	0 (0)	0.999	>99.00	<0.01–>99.00
	Yes	37 (43)	6 (100)			
**Early onset leukopenia**	No	74 (86)	3 (50)	**0.037**	6.16	1.11-34.18
	Yes	12 (14)	3 (50)			
**Thrombocytopenia G3/4**	No	80 (93)	6 (100)	0.999	<0.01	<0.01–>99.00
	Yes	6 (7)	0 (0)			
**AST/ALT G3/4**	No	75 (87.2)	5 (83.3)	0.786	1.36	0.14-12.78
	Yes	11 (12.8)	1 (16.7)			
**Anemia G3/4**	No	81 (94.2)	6 (100)	0.999	<0.01	<0.01–>99.00
	Yes	5 (5.8)	0 (0)			
**Adverse events G3/4**	No	46 (53.5)	0 (0)	0.999	>99.00	<0.01–>99.00
	Yes	40 (46.5)	6 (100)			
**Therapy interruption**	No	61 (70.9)	4 (66.7)	0.999	<0.01	<0.01–>99.00
	Yes	25 (29.1)	2 (33.3)			

*Odds ratios and 95% confidence intervals were calculated using logistic regression.

ALT, alanine aminotransferase; AST, aspartate aminotransferase; CI, confidence interval; NUDT15, nucleoside diphosphate-linked moiety X-type motif 15; OR: odds ratio.The bold values indicate statistical significance (*P*<0.05).

In contrast, there was no statistically significant association between the genetic variants and other 6MP-induced severe toxicity (**Table 6**).

Twenty-seven patients (29.35%) needed 6MP interruption due to infections but there was no significant association between the genetic variants and 6MP interruptions due to infections (*P*=0.999) (**Table 6**).

## 4 Discussion

This study assessed the association of the *NUDT15* (c.415C>T; rs116855232) polymorphism with 6MP dose intolerance in Syrian patients with childhood ALL. We have shown that the evaluated SNP is not common in this population, and that the 6MP dose intensity was considerably lower in the TT genotype than in the CT and CC genotypes.

Pharmacogenetics has contributed to explaining the molecular mechanisms that lead to the interindividual variation in drug response. Thus, it is widely considered as an essential part of personalized medicine. Pharmacogenetics provides a significant benefit in hematology-oncology because of the narrow therapeutic index of chemotherapy drugs and the high incidence of life-threatening AEs. 6MP is the backbone of maintenance chemotherapy in ALL protocols in children. The *NUDT15* polymorphism was confirmed to be associated with 6MP toxicity in multiple populations (13). A few studies have investigated this association in Middle Eastern or Arab children with ALL (10, 23).

Accordingly, the Clinical Pharmacogenetics Implementation Consortium (CPIC) is considering integrating *NUDT15* preemptive genotyping into its current recommendations on thiopurine dosing. Depending on each individual’s *NUDT15* allele combination, *NUDT15* genotypes are classified into three distinct phenotypes. These are poor metabolizers (PMs), intermediate metabolizers (IMs), and normal metabolizers (NMs). The recent CPIC Guidelines for thiopurine categorize an individual harboring one normal function allele plus one NUDT15*3 allele as an IM, while an individual harboring two no-function alleles as PM (24).

The minor allele frequency (MAF) of the *NUDT15* rs116855232 genotype was 3.8% in our population, which is higher than the MAF in the neighboring countries, as it was 0.4%, 0.6%, 1.8%, and 0% in Lebanese, Jordanian, Saudi, and Iranian Kurdish populations, respectively (10, 23, 25, 26). The differences in the frequencies of *NUDT15* polymorphism between the neighboring populations could be attributed to demographic or genetic diversity. Moreover, allele frequency varies among other ethnic groups from 2 to 16%. The frequency of *NUDT15* polymorphism in Chinese patients with ALL is 15.7%, 16% in Japanese, and 11.6% in Taiwan Chinese patients (13, 27, 28). In other populations, the risk allele is less common, occurring at 2% in an admixed American (8), 8.8% in Uruguayan (29), and 5% in Thais (30).

The tolerated 6MP dose in our cohort was 54.69% of the planned dose when there was a *NUDT15* heterozygous (CT) genotype, which is similar to the tolerated dose in the St Jude cohort (63%) (11), lower than Chinese (83.83%) and Japanese (73.2%) (27, 28) but higher than the tolerated dose in the Lebanese cohort (33.3%) and Thai (48.8%) (10, 30). The high percentage of our population compared with the neighboring countries can be explained by the low frequency of the variant in the Lebanese cohort. There was no CT or TT genotypes in the Kurdistan cohort. In case of the homozygous (TT) genotype, the tolerated 6MP dose in our cohort was significantly low (5%), which is close to the tolerated dose in the St Jude study (8.3%) (11) but significantly lower than the tolerated dose in Chinese (60.27%), Japanese (20%), and Thai (16.6%) populations (27, 28, 30), and there was no TT genotype in the Lebanese cohort. Differences between tolerated doses between different populations could be explained by the presence of other genetic/environmental factors that might affect the absorption, metabolism, or excretion of 6MP.

Although the *NUDT15* c.415C>T polymorphism is a risk factor for early onset leukopenia during maintenance therapy for children with ALL (31, 32), *NUDT15* polymorphism was not associated with hepatotoxicity in our cohort; which is similar to the studies by Tanaka et al. (27), Zhou et al. (28) and Moradveisi et al. (23) in a Japanese, Chinese, and Middle Eastern cohorts, respectively. Hepatotoxicity might have other factors that play a role in cooperation with *NUDT15*.

Studying the frequency of polymorphisms of the *NUDT15* not only helps in the dosing of 6MP in patients with ALL, but it may also give an idea of its frequency in patients with inflammatory bowel diseases (IBD) and rheumatoid arthritis to avoid thiopurine-induced toxicity. However, Studies on patients with the other diseases are needed to determine the exact frequency and impact.

There are some limitations to our study. First, due to the small sample size (n=92) and retrospective design of this study, the power to detect differences between small groups is low. Second, the treatment protocols used were not homogenous, which might affect other factors linked to the same toxicity output we looked for. Third, the present study examined the most clinically relevant allele of *NUDT15* and did not investigate all variants. Furthermore, according to the US Food and Drug Administration-approved label for 6MP, patients with severe myeloid toxicity are advised to be tested for TPMT or NUDT15 deficiency, and PMs for TPMT or NUDT15 are recommended to reduce the 6MP dose (33); this study did not examine other genes encoding enzymes of 6MP metabolism, such as *TPMT* and inosine triphosphate pyrophosphatase (*ITPA*). The limitations of our study are partially related to the limited resources and financial support available in our low-income country (Syria) and because of this, we did not examine a larger sample size or examine another gene involved in 6MP metabolism. Therefore, these results should be cautiously interpreted.

In conclusion, our study found that the *NUDT15* polymorphism (c.415C>T; rs116855232) is associated with mercaptopurine-induced early-onset leukopenia but not with hepatotoxicity in a population of Syrian children with ALL. It appears that *NUDT15* polymorphism (c.415C>T; rs116855232) is slightly more frequent in our population than in neighboring countries. The dosage of mercaptopurine should be adjusted based on the genotype of *NUDT15* in patients with ALL.

## Data availability statement

The datasets presented in this article are not readily available because of ethical approval restrictions, this article’s genomic sequencing data cannot be deposited in a public database. Requests to access the datasets should be directed to the corresponding author.

## Ethics statement

The study protocol was approved by the Institutional Review Board of Damascus University, and the committee’s reference number was 514 on December 1, 2019. All procedures involving human participants were performed under the ethical standards of the institutional and/or national research committee and in accordance with the 1964 Helsinki Declaration and its later amendments or comparable ethical standards. As per the ages and conceptual abilities of the patients, informed consent was obtained either from the patient's parents or guardians or from the patients themselves.

## Author contributions

MM: Conceptualization, Data curation, Formal analysis, Funding acquisition, Investigation, Methodology, Software, Writing – original draft, Writing – review & editing. MS: Conceptualization, Methodology, Project administration, Supervision, Validation, Writing – review & editing. MaA: Conceptualization, Methodology, Resources, Software, Supervision, Validation, Writing – review & editing. MoA: Conceptualization, Data curation, Formal analysis, Software, Writing – original draft, Writing – review & editing. KG: Conceptualization, Data curation, Methodology, Resources, Supervision, Writing – review & editing.
